# Spotibot: Rapid scoring of *Botrytis* lesions on rose petals using deep learning and mobile computing

**DOI:** 10.1016/j.plaphe.2025.100029

**Published:** 2025-03-19

**Authors:** Dan Jeric Arcega Rustia, Maikel Zerdoner, Manon Mensink, Richard GF. Visser, Paul Arens, Suzan Gabriëls

**Affiliations:** a Greenhouse Horticulture and Flower Bulbs Business Unit, Wageningen Plant Research, Wageningen University & Research, 6708 PB, Wageningen, the Netherlands; b Plant Breeding, Wageningen University & Research, 6708 PB, Wageningen, the Netherlands; c Graduate School Experimental Plant Sciences, Wageningen University & Research, 6708 PB, Wageningen, the Netherlands; d Food & Biobased Research, Wageningen University & Research, 6708 WG, Wageningen, the Netherlands

**Keywords:** Deep learning, Plant phenotyping, Botrytis, Rose, Mobile computing

## Abstract

Roses are renowned for their ornamental value and are available in a wide range of colors and shapes due to extensive breeding and ease of hybridization. During post-harvest, roses are highly susceptible to fungal decay by the grey mould fungus *Botrytis cinerea*. No complete resistance to *Botrytis* is known, and several studies indicate a quantitative nature of resistance. This implies that multiple genes are involved, and that each contribution may only have a slight effect on resistance. Accurate, fast, and objective phenotyping discriminating between minor effects would be essential for breeding selections and discovering novel resistance- or susceptibility genes against *Botrytis*. Spotibot, a phenotyping software available both as a web application and mobile application, utilizes deep learning and mobile computing for automatically detecting *Botrytis* lesions on rose petals making it highly applicable for breeding selection. The algorithm can measure petal area (mm^2^), lesion area (mm^2^), lesion diameter (mm) and lesion to petal ratio. The deep learning-based algorithm features a coarse-to-fine segmentation approach using two instance segmentation models. The first model (*F*_1_-score ​= ​0.99) detects and segments each petal, while the second model (*F*_1_-score ​= ​0.96) detects and segments *Botrytis* lesions on each petal. Spearman Rank correlation analysis showed a high near-monotonic relationship between human-assessed subjective scores and the objective data generated using Spotibot. An analysis of variance indicated that objective variables reveal more and stronger differences between rose genotypes than using subjective data alone. This is the first work on developing a fast and user-friendly application for image analysis of rose petals to screen *Botrytis* resistance and susceptibility.

## 1 Introduction

Rose is one of the most well-known ornamental crops in the world and is renowned for its elegant flowers, fragrant oils and usage in drinks and food. In 2022, the worldwide production of cut roses exceeded $3B, with the Netherlands as its top exporter ($900M) and the United States as its top importer ($713M) [1]. Roses are of the *Rosa* genus, part of the Rosaceae family which includes many well-known fruit, nut, and flower crops (e.g. rose, apple, almond, and strawberry) [2]. By history, rose cultivars are divided as "Old Garden Roses" and "Modern Roses", of which the modern hybrid tea rose is the most popular and well-known. Furthermore, roses are a species with a relatively small genome size of 500–750 ​Mb. They are known to be diverse and genetically complex due to high heterozygosity and differing levels of ploidy, making precision breeding a difficult task [3].

*Botrytis cinerea,* also known as grey-mould, is one of the main post-harvest fungal pathogens, causing detrimental damage in rose [4]. *Botrytis* is a necrotrophic fungus with a pathogenic lifestyle that kills its host for nutrition. It is a polyphagous pathogen that has a broad host range up to 1400 confirmed plant species, targeting most known flowers, fruits and vegetables [5,6]. Infections on rose petals are commonly observed as necrotic brown expanding lesions which develop aerial grey mycelium over time. This aerial mycelium contains the conidiophores, harboring conidia which are the asexual reproduction spores of *Botrytis*. Conidia spread rapidly through rain, wind and insects, and have a significant presence in the natural environment [7,8]. *Botrytis* can infect roses at all stages of production, including seedling-, growing- and post-harvest stages. Infections are frequently overlooked, as *Botrytis* has a quiescent phase that breaks under yet unknown circumstances [9]. Infections are most severe at the harvest- and post-harvest stage, especially when conditions are moist and the roses are nearing a senescent stage [10].

Breeding is a process in which selected parental plants are crossed to obtain progeny that have a combination of the most favorable traits from their parents. To achieve this, a thorough selection process is required to ensure the best progeny with top-quality traits is selected. This is often a long process that can take 7–20 years to complete for each new rose cultivar [11]. Breeding for resistance is often done by finding a “wild” species with no or little sensitivity towards a pathogen, which subsequently is used in recurrent backcrosses of elite cultivars that carry all desired production and consumer traits absent in the wild species [11,12].

Currently, there is no strong resistance known against *Botrytis* in roses. Genetic association studies suggest that the resistance against *Botrytis* is quantitative, and to date, only a few consistent and relatively large QTL regions have been found through forward genetic approaches in other crops [[13], [14], [15]]. Due to this complex and quantitative nature, it is essential that robust and reliable objective data is generated to aid in the genetic discovery of resistance towards *Botrytis*. In breeding, disease scoring is generally conducted in a subjective manner using a predetermined classified disease scale. However, the phenotypic data required to unravel complex quantitative genetics should be high-resolution and objective. Using image analysis software e.g. ImageJ, it is possible to retrieve the objective lesion parameters related to *Botrytis*. Image analysis using ImageJ is a potentially effective tool for detecting plant diseases, but it requires adequate time to tune numerous image analysis settings [16]. This is relatively challenging for *Botrytis* lesion detection, as roses are extremely diverse in color, which necessitates color-specific image analysis parameters. Therefore, more advanced and automated forms of phenotyping are required for rapid image analysis in breeding trials.

There have been several proposed automated methods for detecting rose *Botrytis* lesions. Most studies involve the use of spectroscopic techniques. For instance, Jafari, Minaee [17] acquired top-facing thermal images of rose plants. They used a radial basis function neural network to classify between healthy and *Botrytis*-infected rose plants using features including thermal histograms, minimum and maximum temperature, and more. Similarly, Ha, Kim and In [18] distinguished between healthy and infected rose plants using thermal images of rose buds and leaves. Their results show that temperature differences as small as 1 ​°C can be used to detect infected rose plants. Meanwhile, Giakoumoglou, Kalogeropoulou [19] have developed an algorithm for the detection of rose *Botrytis* lesions on multi-spectral images of rose leaves. Their algorithm utilizes different deep learning segmentation architectures for measuring lesion area with an accuracy of 90 ​%. All the mentioned studies are helpful for the early detection of rose *Botrytis* infections. However, they are all focused on pre-harvest detection of *Botrytis* and are not designed for lesion quantification in large-scale breeding trials. Furthermore, these techniques are not implemented at breeding companies due to equipment costs, technical experience requirements, and practical limitations. Therefore, a more straightforward approach by red-green-blue (RGB) imaging via mobile phone and deep learning-based detection of *Botrytis* infection on harvested roses is presented in this study.

The use of RGB imaging in plant phenotyping is often coupled with deep learning. Nowadays, deep learning is a highly recommended tool for plant phenotyping because of its flexibility and robustness in analyzing diverse sets of images. By the time of this writing, there is not yet a deep learning algorithm developed for rapid screening of rose *Botrytis* lesions on petals using RGB images. One of the similar and early studies on rose *Botrytis* lesions was done by Cuervo-Bejarano and Lopez-Espinosa [20]. Using a custom lighting environment, the authors acquired images of rose leaves using an RGB camera. They converted the images to the hue-saturation-value (HSV) color space and manually selected regions of interest inclusive of *Botrytis* lesions. Osuna-Caballero, Olivoto [21] developed an RGB-image-based algorithm for segmenting rust disease on pea leaves. Although not applied to roses, their work has proven that RGB imaging can be easily used to quantify plant disease infection areas. Shoaib, Shah [22] have used deep learning to segment plant disease lesions on the so-called Plant Village benchmark dataset. However, the above-mentioned approaches do not involve deployment. Deployment involves integrating an algorithm into a specific user application [23]. Besides setting up a simple approach using RGB imaging, this research focuses on algorithm deployment as an essential part of standardizing and promoting objective plant phenotyping for breeding purposes.

The general objective of this work is to develop a method for rapid and automated screening of rose *Botrytis* lesions to aid in the search for increased resistance during breeding. The specific objectives are as follows: 1) to design a protocol for preparing rose petals for *Botrytis* screening, 2) to develop an algorithm for rapid and accurate image-based quantification of *Botrytis* lesion parameters, and 3) to integrate the algorithm into a mobile application for ease-of-use screening of rose *Botrytis*. This work demonstrates that plant phenotyping can be easily done even using simple equipment, such as a mobile phone, to achieve harmonization and standardization in plant phenotyping.

## 2 Materials and methods

### 2.1 Experimental design

In total, 212 rose cultivars (Rosa spp.) were delivered in batches from five rose breeding companies at five independent locations in Kenya and the Netherlands. The companies involved were Dümmen Orange (DO), De Ruiter Innovations (DRI), Interplant (INT), Meilland International (MI) and United Selections (US). Four companies including DO, DRI, MI, and US grew roses in (polythene-covered) greenhouses in Kenya, while INT cultivated roses were grown in a climate-controlled greenhouse in the Netherlands (20 ​°C and 75 ​% RH). Roses were delivered in 2023 from weeks 16–50, in which each company delivered 40–43 unique genotypes three times at an eight-week interval. Each company had five controls included in each delivery, which were the INT cultivars Bahama, Explorer, Discovery, Royal Class, and Tapdance. Flowers were sprayed with pesticides up to three days before harvest. When the roses reached flowering stage 2 (about to open), the roses were harvested having a stem of approximately 50–60 ​cm and subsequently hydrated on plain water for 24 ​h at 4 ​°C. The harvested roses were transported to Wageningen University & Research in dry and cool (3–8 ​°C) conditions. The INT roses grown in The Netherlands were stored for four days in the same dry and cool conditions to simulate transport from Kenya. After arrival, the rose stems were cut 1–2 ​cm and rehydrated for at least 24h at 4 ​°C. Before performing the disease assay, the roses were climatized for 24h at 20 ​°C and 60 ​% relative humidity in the dark.

*Botrytis cinerea* isolates (B05.10, M3a, BC12, BC25) were cultured on Potato Dextrose Agar (PDA) for a duration of 14 days at an ambient temperature of 20 ​°C under dark conditions. As soon as the mycelium reached the edge of the PDA plate, a 24h UV treatment was done to stimulate *Botrytis* sporulation. To harvest spores, the surface of the PDA plate was rinsed with dH_2_O, and the mycelial debris was removed using a fine sieve. The spore concentration was measured with a hemocytometer and subsequently diluted to a final concentration of 3 x 10^5^ spores/mL with dH_2_O containing 0.01 ​% (V/v) Tween-20 surfactant. The mock treatment had the same composition as the *Botrytis* inoculum but without the fungal spores. The *Botrytis* inoculum was freshly prepared on the same day as the disease assay was conducted.

To discriminate in *Botrytis* sensitivity of the rose cultivars, disease assays were performed on rose petals. Six roses per genotype were used, in which petals were randomly picked from whorls 2–5 excluding the exterior layer of petals and the (closed) flower core. Nunc-bioassay dishes were used with blue wet filter paper and a label at the top right corner including a 2 ​cm-by-2 cm QR code revealing the C-GN-R-AD code where C is the company name, GN is the genotype number, R is the replicate code, and AD is the assay date (Year/Month/Day) e.g. INT-01-A-20230501. In each bioassay dish, 16 petals were laid out with the abaxial side facing upwards. The upper left petal was always given mock treatment (2 ​μL droplet without spores) and turned 45°. All other petals received a 2 ​μL droplet of *Botrytis* (B05.10) inoculum on the center of the petal surface without inducing damage. Per disease assay, each genotype was screened in two independent dishes labeled A or B, and 15 inoculated petals per dish. Furthermore, assays were performed with three biological replicates throughout the year (per genotype *n* ​= ​90). Similarly, disease tests were performed to study variation between *Botrytis* isolates (B05.10, M3a, BC12, BC25). For this, a balanced selection of 19 extremely sensitive or non-sensitive rose genotypes was made. These 19 genotypes were screened with each isolate inoculated independently on four separate petals per bioassay dish combining the test of all 4 isolates in the same dish, in three repeated bioassay dishes (per isolate *n=*228).

*Botrytis* disease progression was measured on the day of inoculation (day 0), and 2-, 3- and 6-days post-inoculation (DPI) by imaging. In addition, subjective disease scoring was done at 3 and 6DPI via a predetermined subjective disease scale from 0 to 5, where 0 ​= ​no lesion and 5 ​= ​capped max lesion score (Fig. S1). The area under the disease progression curve (AUDPC) was generated for each petal, as shown in Eq. (1) [24]. In this equation, the variable *y* represents the *Botrytis* severity score observation at a specific DPI, while *t* denotes the corresponding number of days that have passed. The *n* variable indicates the total number of observations recorded.

(1) $$AUDPC=\sum_{i=1}^{n-1}\frac{\left(y_{i}+y_{i+1}\right)}{2}\times \left(t_{i+1}-t_{i}\right)$$

### 2.2 Image acquisition

**Fig. 1:**
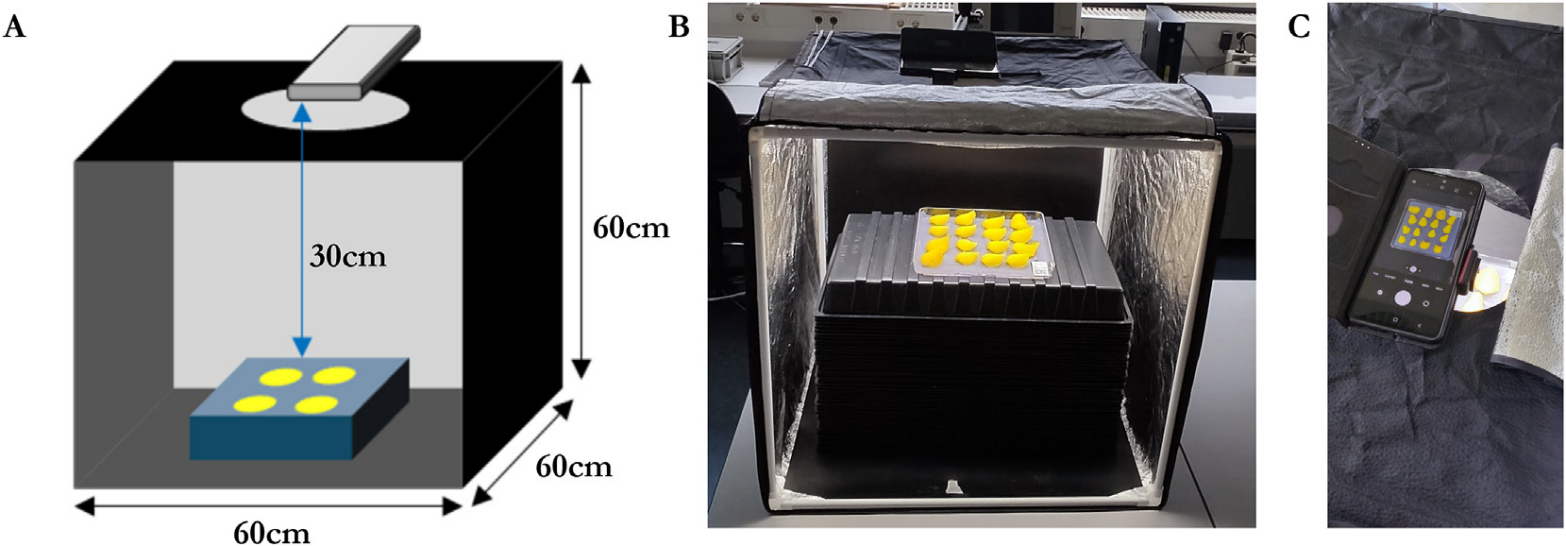
Image acquisition setup: (A) 3D schematic diagram; (B) View inside the photo box; and (C) View seen from the attached mobile phone.

Each topside photo, which shows an entire dish with 16 rose petals, was taken in a Yorbay Photo Studio (60 x 60 ​× ​60 ​cm), as shown in Fig. 1A. Two mid-end mobile phones were used for taking images including Samsung Galaxy A52 (CPU: 2 ​× ​2.2 ​GHz ​+6 ​× ​1.8 ​GHz, Camera resolution: 64 megapixels, Aperture: f/1.8, Focal length: 26 ​mm (wide), Sensor size: 1/1.7″, Pixel size: 0.8 ​μm), and Xiaomi Poco X3 Pro (CPU: 1 ​× ​2.96 ​GHz ​+3 ​× ​2.4Ghz ​+4 ​× ​1.8 ​GHz, Camera resolution: 48 megapixels, Aperture: f/1.8, Focal length: 26 ​mm (wide), Sensor size: 1/2.0″, Pixel size: 0.8 ​μm). Each picture was taken at a distance of 30 ​cm from the Samsung Galaxy A52 phone to the dish, and 40 ​cm from the Xiaomi Poco X3 Pro phone. These distances ensured the phone camera pixel space was fully utilized without zooming (Fig. 1B and C). For both phones, all the camera settings (ISO, shutter speed, white balance, and focus) were set to “auto” to enable optimization of the trained models considering the differences between various mobile phone camera specifications. In both mobile phones, the “full” resolution was used to ensure visibility of all relevant parts of the images, including the dish, petals, ruler, and QR code. The built-in LEDs of the photo studio were set to maximum light intensity.

### 2.3 Dataset distribution

The acquired images were split into two datasets: rose petal dish and rose petal. The rose petal dish dataset is composed of images in which all the petals, the dish, a QR code, and a ruler, were visible; its information is shown in Table 1. Each rose petal dish image was annotated using polygons, with only one class, which is the *petal* class, with the support of the 10.13039/501100000595Darwin v7 annotation platform.

**Table 1:** Rose petal dish dataset information.

Imaging device	Number of images	Image resolution	Number of petal instances	Min. petal height	Max. petal height	Min. petal width	Max. petal width
Samsung Galaxy A52	251	4624 x 3628 px	4845	24	43	761	749
Xiaomi Poco X3 Pro	213	4000 x 1800 px	5068	43	48	520	512

The rose petal dataset is composed of single rose petal images collected in two ways. First, the annotations from the rose petal dish were used to crop each rose petal image. Secondly, the trained instance segmentation model from the first step was used to automatically crop additional rose petal images using its predictions. The background of each rose petal image was removed and the resulting image was rescaled to a resolution of 640 x 640 px. A summary of the dataset is shown in Table 2. Each petal image was annotated using polygons, with only one class, which is the *Botrytis* class, with the support of LabelMe annotation software. The petal images were separated by color as judged by human eye.

**Table 2:** Rose petal dataset information.

Petal color	Number of *Botrytis* instances
Orange	783
Pink	4954
Red	2087
White	1183
Yellow	1248

As a data-centric means of algorithm optimization, the number of petals per color was controlled. As a preliminary test, 500 petal images of each color were annotated and used for training an instance segmentation model. However, it was immediately observed that the model performed relatively poorly on pink, red, and orange petals. Therefore, more petals of the three colors were annotated and used for training, subject to the availability of images. The number of orange petal images was limited since most of the roses provided by the companies were pink, red, and white, reflecting the distribution of the most sold roses in he Netherlands [25]. As a result, the number of orange, pink, and red -colored petals was maximized, while adding some white and yellow -colored petals to balance the model's performance.

All datasets were split according to a 70:20:10 train, validation, and test ratio, in which all the testing results were obtained using the test set. The test set is provided in a public link: https://figshare.com/articles/dataset/Spotibot_Rose_/28163228.

### 2.4 Rose *Botrytis* lesion detection and segmentation algorithm

**Fig. 2:**
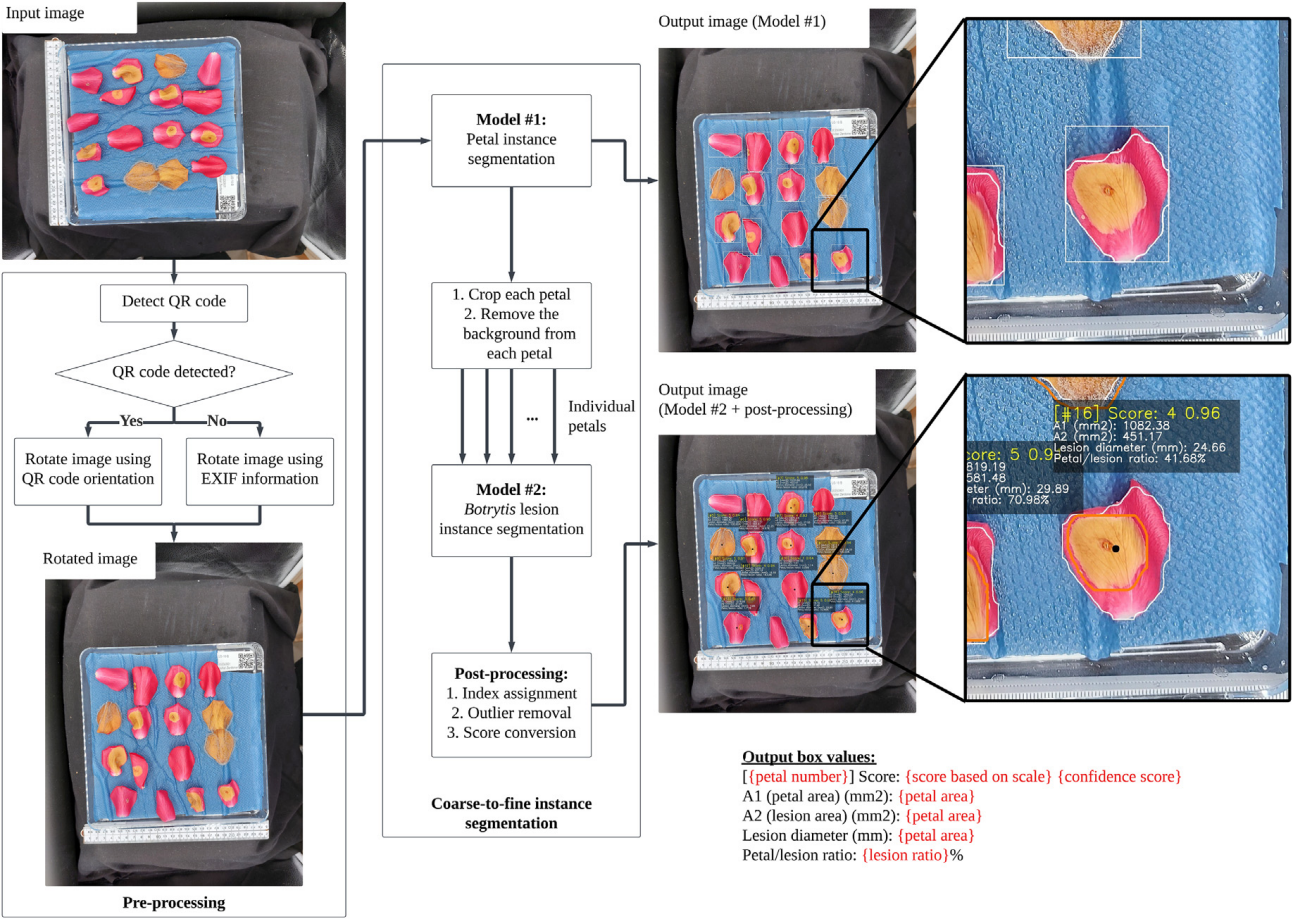
Rose *Botrytis* lesion detection and segmentation algorithm pipeline. Each output box includes several output values as described on the lower right of the figure, where each red-colored text represents an output value. The orange line on each detected object indicates *Botrytis* lesion instance segmentation results and each white line indicates petal instance segmentation results.

The proposed algorithm follows a coarse-to-fine approach, composed of two instance segmentation models, and a post-processing step for assigning indices, removing outliers, and data conversion, as graphically described in Fig. 2. The algorithm was developed using Python programming language (version 3.8.3), with the support of OpenCV image processing library (version 4.10.0) [26], PyTorch deep learning library (version 1.12) [27], and Ultralytics deep learning library (version 8.2.42) [28]. This section describes the algorithm dataset and theoretical considerations.

#### 2.4.1 Pre-processing

Since each trial performed in this work is time-dependent, it is necessary to standardize image orientation, assign correct petal numbers, and associate each detected rose lesion to the corresponding petal. This was achieved by using information from the QR code within each image. First, the correct orientation information from the QR code was scanned using QReader (version 3.12) [29] and rotated as required to ensure consistent indexing among each rose petal. In case a QR code was not detected, the internal image exchangeable image file (EXIF) information was used for proper rotation. The rotated image was then used as input for coarse-to-fine instance segmentation. Additionally, the filename of each image was automatically assigned using the QR code information.

#### 2.4.2 Coarse-to-fine instance segmentation

The main motivation for applying a coarse-to-fine instance segmentation approach instead of a single-step instance segmentation approach is to increase the visibility of small *Botrytis* lesions. It was found from prior testing that a single-step instance segmentation approach was unable to reliably detect small *Botrytis* lesions, which were quite relevant in the conducted trials since such lesions indicate early disease susceptibility at early time points and disease resistance at late ones. The coarse-to-fine instance segmentation approach first detects the rose petals, while *Botrytis* lesions are detected on each rose petal, as shown previously in Fig. 2. To achieve fast model inferences for deployment in mobile phones and good overall performance, You Only Look Once (YOLO) was used as instance segmentation model.

Two versions of YOLO were tested: YOLOv8 [30] and YOLOv9 [31], both maintained by Ultralytics. The two versions were selected due to their suitability for mobile application use; one requirement of our proposed mobile application is that it should run offline and without an internet connection due to poor network connectivity in some remote or strictly controlled environments. YOLOv8 is based on YOLOv5 and is composed of three components: backbone, neck, and head. The backbone utilizes cross-stage partial networks for image feature extraction while the neck combines the extracted features. YOLOv8 uses an anchor-free detector head, which allows it to predict bounding boxes and class probabilities for each object without predefined anchor box sizes [30]. To perform segmentation, a fully convolutional network layer is added to the YOLOv8, calling it YOLOv8-seg. YOLOv9 differs from YOLOv8 due to its additional features including Programmable Gradient Information (PGI) and Generalized efficient Layer Aggregation Network (GELAN). The PGI allows YOLOv9 to retain information during detection, which allows model performance improvement, while GELAN improves the flexibility of YOLOv9. The model input size for both petal instance segmentation (model #1) and *Botrytis* lesion instance segmentation (model #2) was set to 640 x 640 px.

#### 2.4.3 Post-processing

By default, the instance segmentation models do not have sorted output results. To standardize the trials, indices were assigned to each petal object detected. The centroid y-coordinate of all detected rose petal objects was sorted in ascending order and stored in a list. Each row was determined using the following process: the first rose petal object in the sorted list acts as a reference object and its centroid y-coordinate was subtracted from the centroid y-coordinate of each remaining rose petal object. If the difference between the centroid y-coordinate of the reference object and the centroid y-coordinate of each rose petal object was lower than 350, it was included in the row, otherwise excluded from the row. The value of 350 px was determined based on approximately half of the maximum width of each petal, as shown previously in Table 1. The centroid x-coordinates of the remaining objects were sorted in ascending order to determine the indices of each object in the specific row. This process continues until no objects are left on the list.

As observed from previous trials, there were occasions where *Botrytis* lesions appeared on the edges of a petal, which were usually caused by cross-contamination or natural infection. Such lesions were excluded since *Botrytis* was applied to the center of each petal and lesions on the edges of a petal did not properly represent the effect of artificial inoculation in the time course. Since plant breeding trials are the main target of the algorithm, only the detected *Botrytis* lesion closest to the centroid of each petal was retained. This was done by programmatically limiting the number of detected *Botrytis* lesions on each petal to 1. Next, the diameter of each detected *Botrytis* lesion was measured and converted to actual objective measurements in mm. A reference distance was manually obtained from the ruler present in each image and the number of pixels across the reference distance was counted. Since the picture shooting distance of the mobile phone was fixed, similar reference distances were used. The reference distance and number of pixels were used to calculate the pixel conversion ratio *c*, as shown in Eq. (2).

(2) $$c=\frac{reference\;distance\;in\;mm}{reference\;\#\;of\;pixels}$$

The pixel conversion ratio was multiplied by the measured pixel width and pixel height to obtain the actual petal and lesion diameter in mm, petal and lesion area in mm^2^, and lesion-to-petal ratio. Each actual diameter was measured using the petal or lesion contours using the rotating calipers algorithm [32], where the computed major axis is the diameter. Petal and lesion area were computed using the definition of ellipse area, as shown in Eq. (3).

(3) $$Petal\;or\;lesion\;area\;\left({mm}^{2}\right)=\pi \left(\frac{petal\;or\;lesion\;width*c}{2}*\frac{petal\;or\;lesion\;length*c}{2}\right)$$

Finally, the lesion ratio was measured by dividing the lesion area over the petal area. However, as a post-processing condition, the lesion ratio was computed as 1 when the lesion area was higher than the petal area since the lesion cannot be larger than the petal area.

#### 2.4.4 Model training and algorithm optimization

Model training and testing were performed using a desktop computer running under Ubuntu 22.04 operating system, with an Intel Xeon E5-1650 processor, NVIDIA GeForce GTX Titan X GPU, and 16 ​GB RAM. For mobile phone testing, both the Samsung Galaxy A52 and the Xiaomi Poco X3 Pro were used for inference time testing. For testing and deployment, all models were converted from uncompressed PyTorch model format to Open Neural Network Exchange (ONNX) format, while using ONNX runtime (version 1.15.0).

For the petal instance segmentation model, model-centric optimization was performed, concentrating on minimizing inference time. Different sizes of YOLOv8 and YOLOv9 were tested, specifically nano (n) and small (s) for YOLOv8, and compact (c) for YOLOv9. Learning rate (*lr*) was iteratively tested, with values including 0.05, 0.01, 0.005, and 0.001. A batch size of 2 and 25 training steps per epoch was used.

For the *Botrytis* instance segmentation model, data-centric optimization and model-centric optimization were both applied. It was mentioned previously in Section 2.3 that the number of annotated petal images were controlled based on petal color. For model-centric optimization, different model sizes were used, similar to the petal instance segmentation model. Different *lr* values of 0.01, 0.005, and 0.001 were used, with a batch size of 16 and 40 training steps per epoch.

#### 2.4.5 Algorithm evaluation

The algorithm was evaluated based on detection and segmentation performance. In both sets of metrics, Intersection over Union (*IoU*) was used for evaluation. *IoU* ranges from 0 to 1.0, where values closer to 1.0 mean better overlap between predicted and ground truth values. For detection, *IoU*_*det*_ was computed as shown in Eq. (4).

(4) $${IoU}_{\det}=\frac{area\left(b_{pred}\cap b_{gt}\right)}{area\left(b_{pred}\cup b_{gt}\right)}$$

If the *IoU*_*det*_ of two compared objects were more than 0.5, then it was considered a match. Each match was classified as true positive (*TP*_*det*_), false positive (*FP*_*det*_), and false negative (*FN*_*det*_). The matches were used to compute precision, recall, and *F*_1_-score, as shown in Eqs. (5), (6), (7)).

(5) $$Precision=\frac{{TP}_{\det}}{{TP}_{\det}+{FP}_{\det}}$$

(6) $$Recall=\frac{{TP}_{\det}}{{TP}_{\det}+{FN}_{\det}}$$

(7) $$F1-score=\frac{2*precision*recall}{precision+recall}$$

On the other hand, *IoU*_*seg*_ is measured by counting the true positive *TP*_*seg*_, false positive *FP*_*seg*_, and false negative *FN*_*seg*_ pixels after matching the predicted and ground truth masks and is computed using Eq. (8).

(8) $${IoU}_{seg}=\frac{{TP}_{seg}}{{TP}_{seg}+{{FP}_{seg}+FN}_{seg}}$$

### 2.5 Spotibot application and usage instructions

**Fig. 3:**
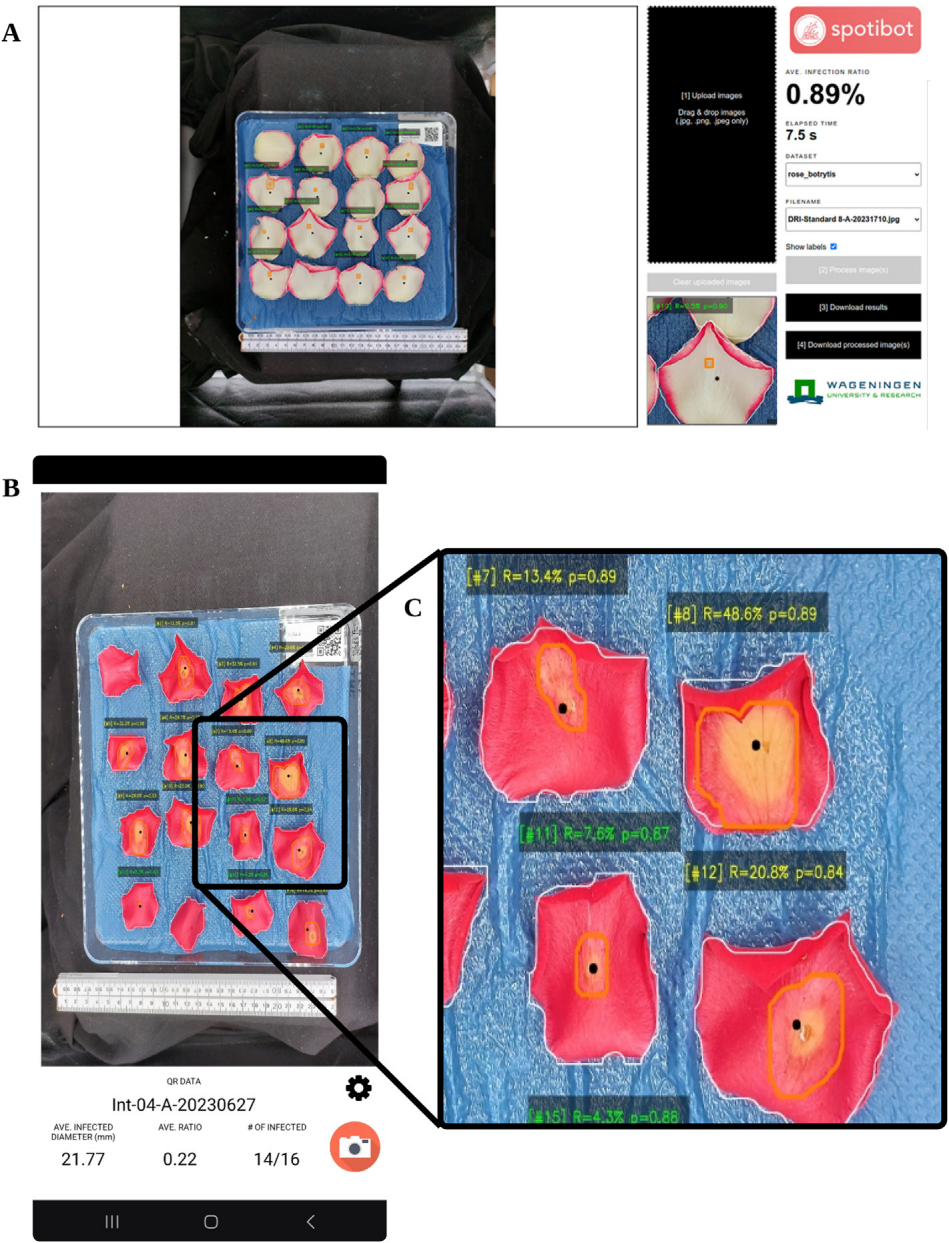
Screenshots of Spotibot: (A) Web application; (B) Mobile application; and (C) Zoomed-in portion of the output image from (B), where R is the lesion and petal ratio, and p is the confidence score.

The algorithm was integrated into a software application called Spotibot, for “Spotting *Botrytis*”. Spotibot is available as a web application and a mobile application, as shown in Fig. 3. The web application was written in Python, with the support of Flask (version 3.0.3). Meanwhile, the mobile application was written in Java. Both applications can be used by request and permission from the authors.

First, the user turns on the LED lights, and then places the bioassay dish inside the photo box. As previously mentioned, the bioassay dish can be placed at an amenable distance to cover most of the visible areas of the mobile phone camera. To ensure that there are no petals missed by the algorithm and to prevent cross-contamination, each petal should be separated from the others. The user attaches a mobile phone to the phone stand, with the rear camera facing the bioassay dish. If using the web application, the user can take images using the default phone camera app, then the images can be uploaded to the web application for processing. The user can download the summary of results in a.csv file and optionally download the processed images. However, one limitation of using the web application is that the user cannot be prompted in case a QR code is not found in an image. The mobile application on the other hand does warn the user if the image was unclear or the QR code was not detected. The approach for downloading files is the same for the web- and the mobile application, being .csv files.

### 2.6 Statistical analysis

A Spearman rank correlation analysis was performed to evaluate the relationship between the subjective scores and objective variables, dependent and independent of color [33]. Furthermore, for each variable, a two-way analysis of variance (ANOVA) was performed to assess significant differences between the genotypes [34]. To further identify differences in genotype pairs, a Tukey's Honestly Significant Difference (HSD) test post-hoc comparison was performed [35]. The threshold for the significance of each analysis was set at *p* ​< ​0.05. The data was normalized (1, −1) for a graphical comparison of subjective scores and objective variables in the controls. All statistical tests were performed using R software (version 4.4.1) [36] and R studio (version 1.4.1717) [37].

## 3 Results

### 3.1 Model optimization

The results of optimizing the petal instance segmentation model and *Botrytis* instance segmentation model using different model sizes are shown in Table 3, Table 4. The results in Table 3 show that the petal instance segmentation model had excellent performances regardless of model size. It was observed that YOLOv8n-seg had the fastest average inference time using the PC and the mobile phones, compared to the other two models, while having a stable performance despite having the least model parameters. Therefore, the YOLOv8n-seg was used for all subsequent analysis and for actual model deployment.

**Table 3:** Petal instance segmentation model training and optimization results.

Model	Best *lr*	*F*_1_-score	Miss rate	Ave. *IoU*_*seg*_	Ave. inference time		
					GPU (PC)	CPU (Samsung Galaxy A52)	CPU (Xiaomi Poco X3 Pro)
YOLOv8n-seg	0.05	0.99	0.001	0.90	571 ​ms	602 ​ms	571 ​ms
YOLOv8s-seg	0.05	0.99	0.002	0.91	604 ​ms	1.32s	1.12s
YOLOv9c-seg	0.01	0.99	0.01	0.90	602 ​ms	3.83s	3.20s

**Table 4:** *Botrytis* instance segmentation model training and optimization results.

Model	Best *lr*	*F*_1_-score	Miss rate	Ave. *IoU*_*seg*_	Ave. inference time		
					GPU (PC)	CPU (Samsung Galaxy A52)	CPU (Xiaomi Poco X3 Pro)
YOLOv8n-seg	0.01	0.96	0.05	0.90	50 ​ms	423 ​ms	211 ​ms
YOLOv8s-seg	0.01	0.97	0.05	0.91	57 ​ms	1049 ​ms	765 ​ms
YOLOv9c-seg	0.001	0.96	0.07	0.90	73 ​ms	1079 ​ms	789 ​ms

The data summarized in Table 4 show that the three optimized models had close performances in *Botrytis* lesion instance segmentation. However, YOLOv8s-seg stood out since it has the highest *F*_1_-score and a relatively low miss rate, but a slightly longer average inference time compared to YOLOv8n-seg upon using the PC. By testing on the mobile phones, it appeared that the YOLOv8n-seg has a much lower inference time than the YOLOv8s-seg. In this step, the inference time is a crucial value, since a long inference time for 16 petals per image will severely impact the speed of the mobile application. Therefore, even though YOLOv8s-seg had a slightly better performance compared to the other two models, YOLOv8n-seg was still selected for deployment since its inference time was very low.

**Fig. 4:**
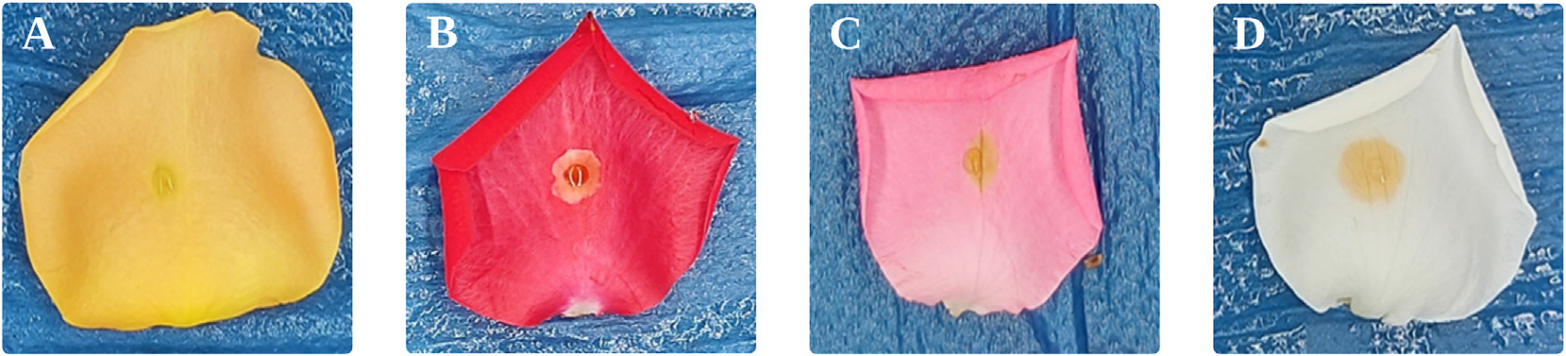
*Botrytis* lesion development on different rose petal colors: (A) Orange petal; (B) Red petal, (C) Pink petal; and (D) White petal.

The data in Table 5 clearly show that the model had more difficulties in detecting *Botrytis* from orange petals. This is likely because orange petals have little discoloration whenever they are affected by *Botrytis*, causing confusion to the model (Fig. 4A). In addition, the model had some error in detecting *Botrytis* from red petals (Fig. 4B) but still showed an excellent performance since *Botrytis* was easily visible. For pink and white petals, the model easily detected lesions because of the high contrast between the color of the lesion and the color of the petals (Fig. 4C and D).

**Table 5:** *Botrytis* instance segmentation model performance based on petal color.

Petal color	*F*_1_-score	Ave. *IoU*_*seg*_	Miss rate
All colors	0.97	0.90	0.04
Orange	0.92	0.84	0.14
Pink	0.99	0.93	0.00
Red	0.96	0.96	0.04
White	1.00	0.88	0.00
Yellow	0.98	0.91	0.02

### 3.2 Speed benchmarking

The total processing time of the algorithm was analyzed by measuring the processing time after each algorithm step, as shown in Table 6. All the results were obtained using the YOLOv8n-seg models for both petal instance segmentation and *Botrytis* instance segmentation, while assuming there are 16 petals on each dish image. It shows that, by using a GPU-equipped PC, all the images (4155 images) can be processed in about 2.7h. When using a mobile phone, it takes about 11.3h using the Samsung Galaxy A52, and 7.2h using the Xiaomi Poco X3 Pro. This analysis does not include bringing the dish in and out of the photo box, nor acquiring the images. Comparing the processing time to the time for manual subjective scoring, it typically takes about 5s (when there are no or full lesions on the 16 petals) to 30s for an experienced person to look at the petals and fill in the scores in a spreadsheet, with a total of about 23h.

**Table 6:** Algorithm speed benchmarking results.

Algorithm step	Ave. inference time		
	GPU (PC)	CPU (Samsung Galaxy A52)	CPU (Xiaomi Poco X3 Pro)
Pre-processing	1030 ​ms	2306 ​ms	2104 ​ms
Petal instance segmentation	571 ​ms	602 ​ms	571 ​ms
*Botrytis* instance segmentation	800 ​ms (50 ​ms x 16)	6768 ​ms (423 ​ms x 16)	3376 ​ms (211 ​ms x 16)
Post-processing (object sorting)	23 ​ms	103 ​ms	87 ​ms
Post-processing (data conversion)	21 ​ms (1.3 ​ms x 16)	35 ​ms (2.2 ​ms x 16)	32 ​ms (2.0 ​ms x 16)
Total	2.44s	9.8s	6.2s
Total (4155 images)	2.7h	11.3h	7.2h

### 3.3 Qualitative results

**Fig. 5:**
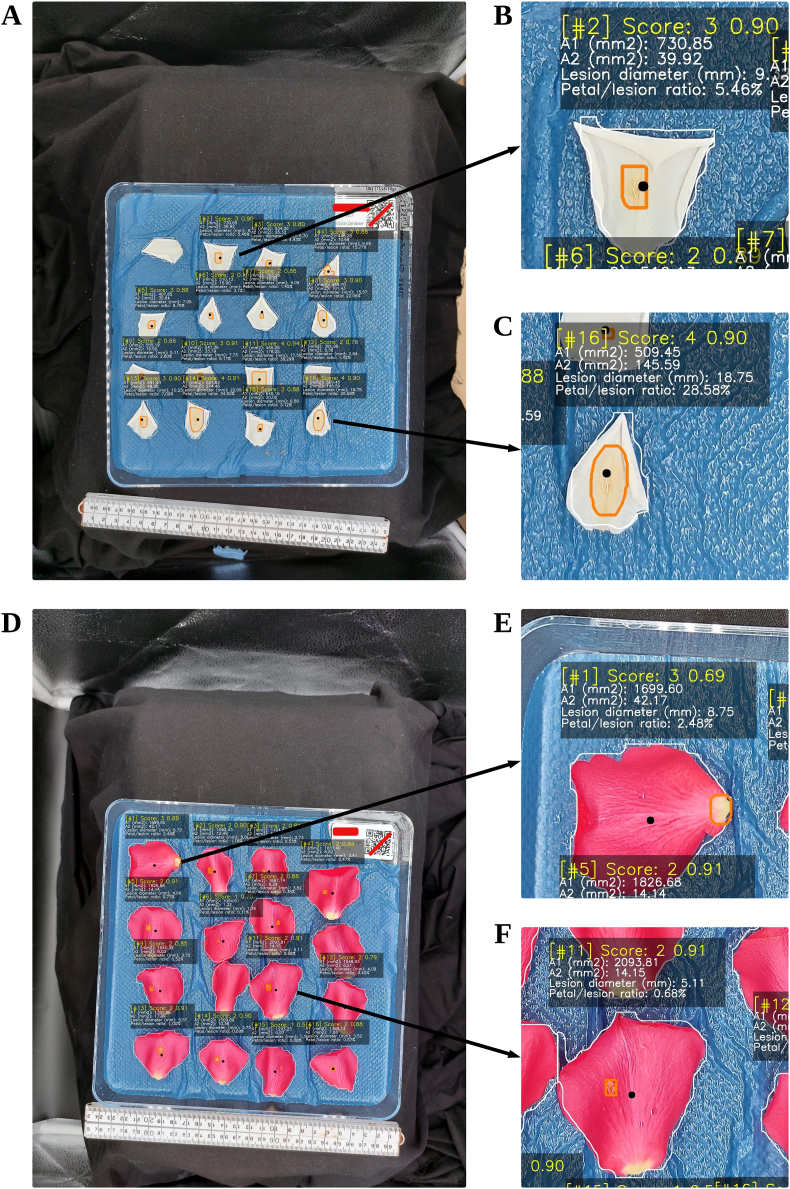
Best algorithm output results: (A) Detections on a dish with white petals while (B and C) shows zoomed in correct output results; (D) Detections on a dish with red petals while (E) shows a sample output error and (F) shows correct output results. The representation of the values in each output box is shown previously in Fig. 2, while the orange line on each detected object indicates *Botrytis* lesion instance segmentation results and each white line indicates petal instance segmentation results.

Sample algorithm output image results are shown in Fig. 5. It can be clearly seen that the algorithm performed well in detecting and segmenting *Botrytis* on white petals (Fig. 5A). However, the petal segmentation results were seldom outside of the petal boundaries (Fig. 5B and C) since the petal objects were quite small relative to the total image dimensions. Since the extra pixels from segmentation did not largely differ from the actual petal size, this issue was considered negligible. For the red petals (Fig. 5D), the detection results show that the algorithm was still able to detect the petals and *Botrytis* lesions but there was a single error in detecting a white part of the petal which was not a lesion (Fig. 5E). But in all other cases, the detection was accurate and produced good segmentation results, as shown in Fig. 5F.

**Fig. 6:**
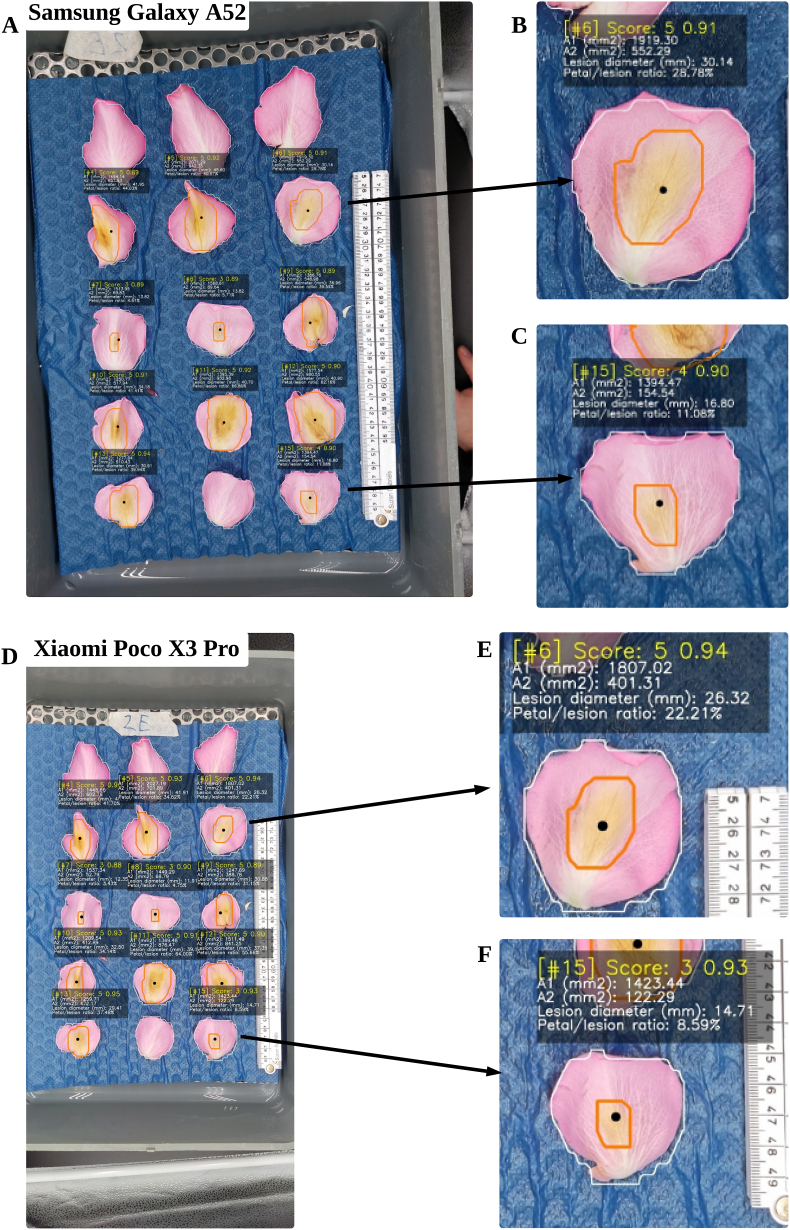
Algorithm output results on different mobile phones: (A, B, and C) Samsung Galaxy A52; and (D, E, and F) Xiaomi Poco X3 Pro, the representation of the values in each output box is shown previously in Fig. 2, while the orange line on each detected object indicates *Botrytis* lesion instance segmentation results and each white line indicates petal instance segmentation results.

The algorithm was also tested on different mobile phones, as shown in Fig. 6. It shows that there were minor differences in the results obtained between the use of the two mobile phones.

### 3.4 Subjective vs. objective data analysis results

#### 3.4.1 Color independent analysis

**Fig. 7:**
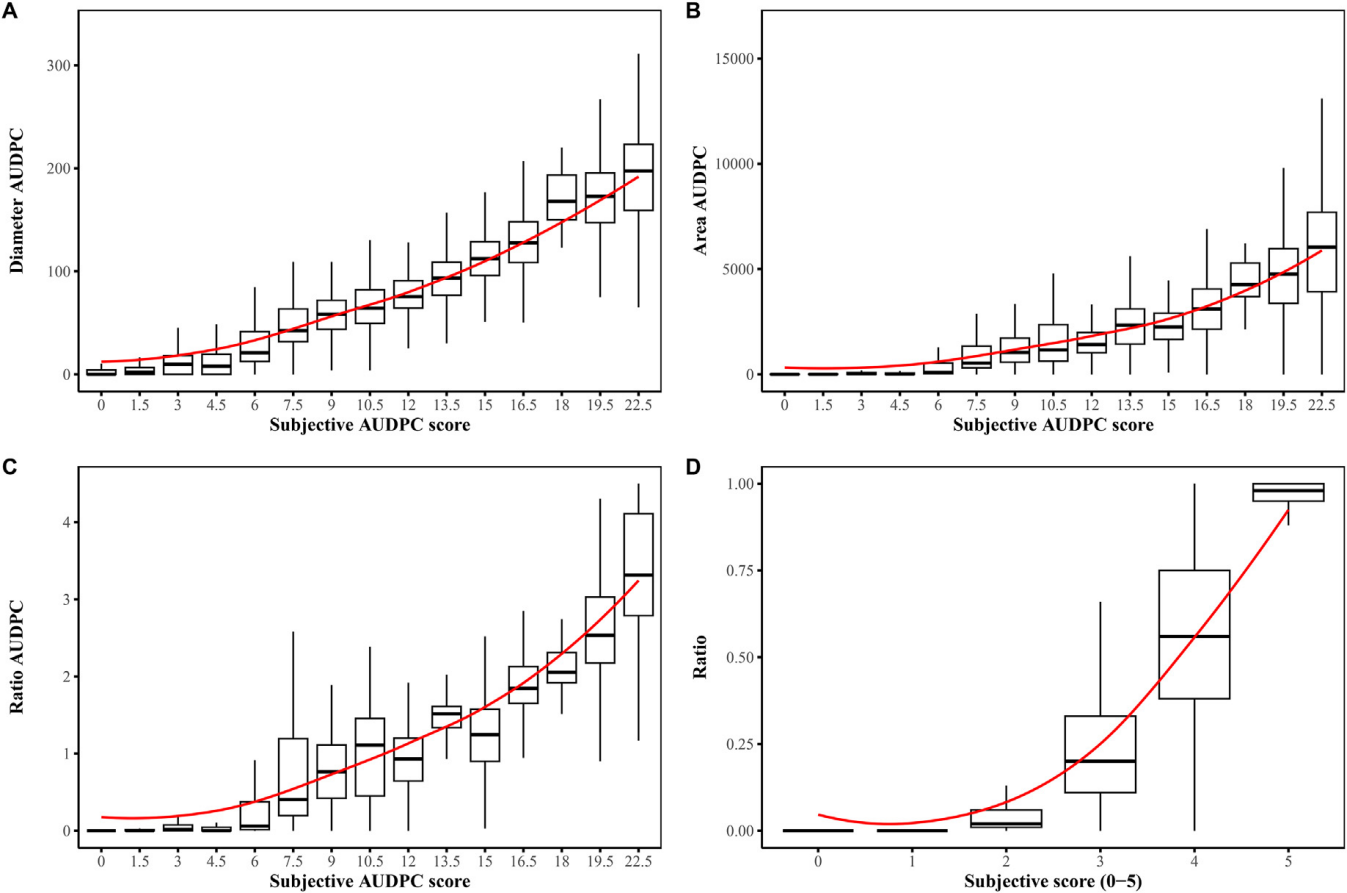
Box plots with trendlines illustrating non-linear positive correlations between subjective scores and objective variables. (A) Diameter AUDPC versus subjective AUDPC score. (B) Area AUDPC versus subjective AUDPC score. (C) Ratio AUDPC versus subjective AUDPC score. (D) Ratio versus subjective score (0–5) at 6DPI. The AUDPC values are a combination of the sensitivity data from 3DPI and 6DPI. The box plot whiskers extend to 1.5 times the interquartile range from Q1 and Q3. The trendlines were computed using locally estimated scatterplot smoothing (LOESS).

To compare subjective and objective data, regardless of color, a Spearman rank correlation (*ρ*) analysis was performed. The results summarized in Table 7 show that the objective scores had a significantly (*p* ​< ​0.01) strong positive correlation with the subjective scores. This indicates a positive, nearly monotonic relationship, between the objective and subjective data, as shown by the increasing trend in Fig. 7. Based on the AUDPC correlations, the strongest relationship between subjective and objective data was found in the ratio (*ρ* ​= ​0.88) variable, followed by the lesion diameter (*ρ* ​= ​0.88) and area (*ρ* ​= ​0.83) variables. For the 3DPI subjective scoring, the objective data were strong and evenly correlated to the subjective data. However, the correlation between ratio scores and subjective scoring at 6DPI was notably stronger. Nevertheless, strong correlations were observed between subjective scores and all objective variables.

**Table 7:** Color independent spearman rank correlation values for subjective and objective scores. All calculated *p*-values were <0.001.

Subjective data	Objective data	*ρ*	*t*-statistic
3DPI score	Diameter	0.85	1.190 ​× ​10^11^
	Area	0.85	1.179 ​× ​10^11^
	Ratio	0.84	1.324 ​× ​10^11^
6DPI score	Diameter	0.77	1.889 ​× ​10^11^
	Area	0.78	1.807 ​× ​10^11^
	Ratio	0.83	1.353 ​× ​10^11^
AUDPC score	Diameter (AUDPC)	0.88	9.953 ​× ​10^10^
	Area (AUDPC)	0.83	1.370 ​× ​10^11^
	Ratio (AUDPC)	0.83	9.588 ​× ​10^10^

#### 3.4.2 Color dependent analysis

To verify if the petal color affected the analysis, spearman rank correlation analysis was also performed per petal color, as shown in Table 8. All *ρ* values indicated that there was a strong relationship with the objective data, regardless of petal color. Generally, the data obtained from the red and pink petals showed the highest *ρ* values to the subjective scores. The data obtained from white and yellow petals also showed high *ρ* values, but the 6DPI data showed lower *ρ* values compared to the 3DPI data. For the orange petals, however, the obtained data had lower correlations, especially for the 3DPI data. This is due to the slightly worse performance of the model on the orange petals, as described previously in Section 3.1. Overall, the results showed that the algorithm was not dependent on color and was able to yield results that were comparable to subjective scoring.

**Table 8:** Color dependent spearman rank correlation values for subjective and objective AUDPC scores. All calculated *p*-values were <0.001.

Petal color	*ρ* (diameter)	*ρ* (area)	*ρ* (ratio)
All colors	0.88	0.83	0.83
Red	0.88	0.86	0.86
Pink	0.86	0.82	0.86
White	0.83	0.76	0.85
Yellow	0.83	0.76	0.85
Orange	0.76	0.73	0.76

#### 3.4.3 Objective data impact analysis

To elucidate the impact of objective data on the statistical comparison of 212 genotypes, a two-way analysis of variance (ANOVA) was conducted (Table S1). For both subjective and objective data, a strong and significant (*p* ​< ​0.001) genotypic effect was observed. The area variable had the highest mean square values and represented the test including the most variation. Although the significance was identical in all tests, the F-values were higher for the objective variables, rather than using the subjective scores in the analysis.

**Fig. 8:**
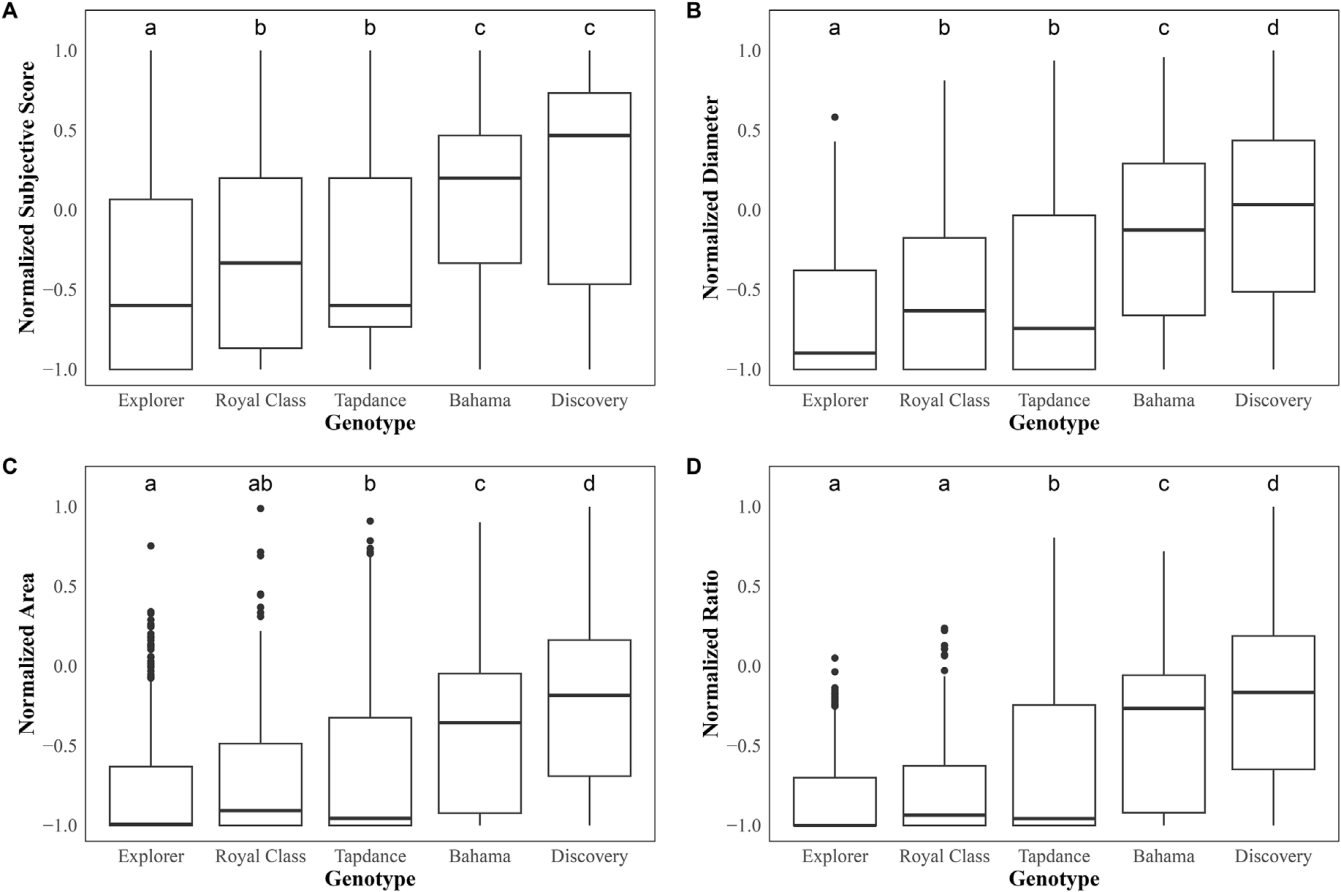
Box plots illustrating comparisons of *Botrytis* sensitivity between rose control genotypes using subjective and objective data. Sensitivity data for (A) subjective score, (B) diameter, (C) area, and (D) ratio are depicted. The y-axis represents AUDPC values, which are a combination of sensitivity data at 3DPI and 6DPI. The data were normalized from 1 to −1 to allow comparison of each variable. The box plot whiskers extend to 1.5 times the interquartile range from Q1 and Q3. Beyond this range data points are considered outliers and are shown as individual black dots. Genotype-genotype pair differences, determined by Tukey's HSD test (*p* < 0.05), are shown as letters above each box to indicate significantly different pairs.

The control genotypes were exclusively analyzed as an example to observe the effect of objective data on measuring subtle differences between the genotypes, as summarized in Fig. 8. Each normalized graph showed a clear distinction between the control genotypes, which are less sensitive to *Botrytis* (Explorer, Royal Class, Tapdance), and the susceptible control genotypes (Discovery, Bahama). The differences between the less sensitive genotypes appeared to be minor. A two-way ANOVA, with each variable, showed comparable results as the statistical comparison of 212 genotypes, with objective data having a large F-value and the area variable specifically having the highest mean square values. A Tukey's HSD test revealed that most genotypes are significantly different from each other (*p* < 0.05).

**Fig. 9:**
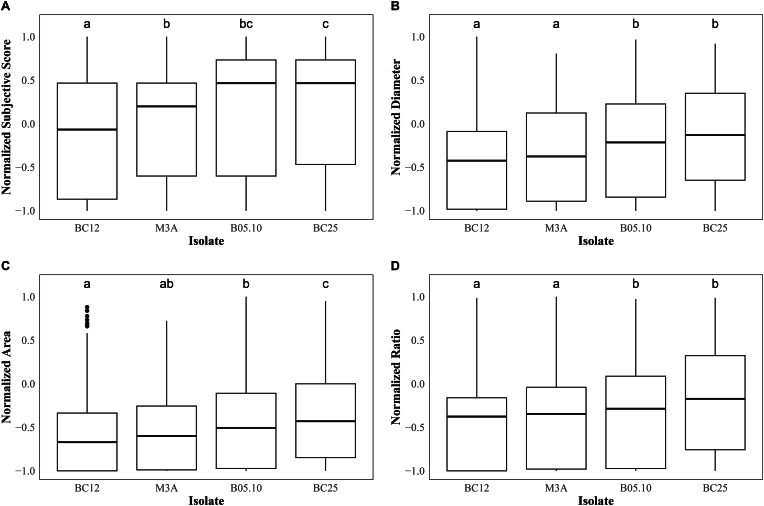
Box plots illustrating comparisons of *Botrytis* isolates on a selected group of genotypes using subjective and objective data. Sensitivity data for (A) subjective score, (B) diameter, (C) area, and (D) ratio are depicted. The y-axis represents AUDPC values, which are a combination of sensitivity data at 3DPI and 6DPI. The data were normalized from 1 to −1 to allow comparison of each variable. The box plot whiskers extend to 1.5 times the interquartile range from Q1 and Q3. Beyond this range data points are considered outliers and are shown as individual black dots. Isolate-isolate pair differences, determined by Tukey's HSD test (*p* < 0.05), are shown as letters above each box to indicate significantly different pairs.

To discover the value of objective image analysis using Spotibot for measuring subtle variation in resistance levels, a selected group of genotypes was inoculated with four different *Botrytis* isolates. Although the differences between resistance levels to the different *Botrytis* isolates were minimal, the order of genotypes from most-to least resistance remains the same in both the subjective as well as objective measurements. A two-way ANOVA with Tukey's HSD test showed differences between the more aggressive isolates (B05.10 & BC25) and the less aggressive isolates (M3A & BC12). Overall, we have observed that, with the objective data from Spotibot, it was possible to rank the resistance levels of genotypes. Furthermore, the objective data enabled discrimination between the most aggressive (B05.10 & BC25) and less aggressive (M3A & BC12) isolates (Fig. 9).

## 4 Discussion

### 4.1 Spotibot accelerated rose *Botrytis* lesion screening with less expert reliance

The Spotibot app has demonstrated the capability to generate reliable and objective screening data with minimal human intervention. Lesion screening is predominantly performed through visual inspection without the aid of measurement tools such as rulers or calipers. This manual approach results in subjective data that often is biased to the observer's expertise, as it lacks precise measurements of parameters like diameter, area, and ratio. The Spotibot algorithm significantly enhances the objectivity and efficiency of this process, reducing labor costs associated with bioassay scoring. However, one source of error identified in the results was the presence of overlapping petals. Despite the algorithm being trained on diverse images, some petals were prepared in a manner that caused partial overlap. This necessitates a visual check of researchers or personnel, when pictures are taken, to ensure petals do not overlap, as this can lead to cross-contamination, which must be avoided throughout the trial.

### 4.2 The algorithm performed well on varying images

One special characteristic of the algorithm is that the accompanying models were not trained on color calibrated images. This allows for a more generalized approach, since not all mobile phones have good automatic color balancing or correction. It also appeared that model-centric optimization, such as by network architecture alteration, was not necessary as the target dataset required a data-centric optimization approach. A foreseeable improvement for the algorithm is to acquire additional images of orange petals to enhance the model's overall performance.

The algorithm had small measurement variations when using different mobile phones. Two mobile phones were compared: Samsung Galaxy A52 and Xiaomi Poco X3 Pro. One major difference between the two mobile phones was their camera resolutions. The camera resolution was important to ensure that all the parts of the image were visible, especially the dish and the petals. The Xiaomi Poco X3 Pro can acquire longer images while the Samsung Galaxy A52 can take slightly wider images. As shown previously in Fig. 6, there were more visible parts of the dish and the surroundings in taking images using the Xiaomi Poco X3, but the results were still highly identical to the Samsung Galaxy A52 which had the samples mostly in sight. The measurements were highly similar although some difference in lesion diameter was detected. This was due to the slight overestimation of the *Botrytis* instance segmentation model in getting the boundaries of the *Botrytis* lesion. In most cases, the lesion diameter difference was small to only about 2 ​mm. Overall, the algorithm output was consistent.

Another difference between the mobile phones was their CPU specifications. The Xiaomi Poco X3 Pro can operate at 2.96 ​GHz, while the Samsung Galaxy A52 can only operate at 2.2 ​GHz. This led to inference time differences between the mobile phones in which Xiaomi Poco X3 Pro was faster.

From these observed differences, the recommended camera resolution is about 48 megapixels and above while it is most important to ensure that all relevant parts of the image are visible by using the “full” resolution settings. In terms of speed, it showed that the mobile application should preferably be used with a CPU frequency equal or faster than 2.2 ​GHz.

Through this work, it was proven that the development of computer vision and AI solutions for plant phenomics has rapidly evolved over the past few years. Before, the use of deep learning models on mobile phones has been problematic due to factors including model complexity, mobile phone performance limitations, and more. Therefore, it is important to acknowledge that the techniques used in this work are based on current state-of-the-art methods that are available during this time. In the future, it might be possible that the processing time of the mobile application can still be improved, which could allow users to instantly see the algorithm output results. But most of all, it shows that more complex plant phenotyping tasks may also be performed using portable devices, such as a mobile phone.

### 4.3 Spotibot allowed better precision for genetic association studies

To enable discoveries of quantitative traits, good quality, and high-resolution data are required. We have shown that objective data from Spotibot is able to increase the quality of statistical analysis over subjective data, resulting in more reliable genotype differences. High correlations were found between subjective and objective data, providing confidence in observations made with Spotibot. Notably, at 6DPI, the ratio variable had a higher correlation with subjective scoring than the other variables. This might be due to the large number of petals fully engulfed with *Botrytis*, which gave maximum (100 ​%) ratio values, as previously seen in Fig. 7D. The subjective assessment was based on a discrete categorical estimation of lesion diameter (Fig. S1). Therefore, it was expected that the diameter variable would correlate best with subjective scores, however, the ratio variable had the strongest relationship at 6DPI. This is possible due to petal size limitations that cap the lesion growth at different diameters, which for the ratio variable is capped at one maximum value similar to the highest subjective score.

Furthermore, the major differences observed between subjective and objective variables were in the genotype-genotype pairs that were significantly distinct. It was notable that with all objective data, it was possible to make a distinction between the susceptible control genotypes, Discovery and Bahama. This might be due to the algorithm's ability to obtain higher precision at the higher scores, while subjective data differences between the pre-determined scales were set and limited at this level. All the variables, except the ratio value, were not able to show a significant difference between Tapdance and Royal Class control genotypes. However, the subjective score and diameter variable showed a significant difference between Royal Class and Explorer, while the ratio and area variables did not. The 1st quartile and median values of the ratio and area variables were low for the less sensitive control genotypes in comparison with the subjective score and diameter variable. This reflected that there was a different level of precision found in the variables at the lowest end of the scoring scale. Rather than using one objective variable, we conclude that it will be most suitable to include all objective variables in genetic association studies, as there might be slightly more or less precision at each end of the disease scale.

### 4.4 Objective data enhances subtle differences between *Botrytis* isolates

While the main aim of Spotibot is to screen rose cultivars for *Botrytis* sensitivity, the tool can also be applied in a similar setup to make additional biological discoveries. We showed that implementing objective data aids in uncovering subtle differences between *Botrytis* isolates. Although there is a high similarity between isolates, a difference in aggressiveness was identified. For isolate M3A and B05.10 a similar difference in aggressiveness was observed by You et al., in tomato [38]. The level of aggressiveness of BC25 was surprising, as it was previously shown to be nearly non-aggressive in tomato [39]. BC25 was isolated from rose [39], thus despite the broad host range of *Botrytis,* this isolate is likely more adapted to Rose cultivars. Oppositely, BC12, which was isolated from gerbera [40], is shown to be less aggressive on roses. This might indicate that isolates from other crops do not directly correlate to aggressiveness on rose and vice versa. Overall, Spotibot data strengthens the confidence of subtle differences between *Botrytis* isolates, compared to results obtained using subjective data. Therefore, Spotibot has shown to be a valuable tool in differentiating between resistant levels of rose genotypes as well as the aggressiveness of *Botrytis* isolates.

### 4.5 Conclusion

In this work, an algorithm for measuring the size of *Botrytis* lesions on rose petals was developed and deployed in a software application called Spotibot. The findings demonstrate that Spotibot is fast, robust, and consistent in detecting *Botrytis* lesions while providing richer information than manual scoring parameters such as lesion diameter, lesion area, and lesion-to-petal ratio. Notably, the data generated by Spotibot strongly correlates with human-assessed subjective scores. While the algorithm performs well across various petal colors, it exhibited varying accuracy for subtle color changes, most especially on orange petals. Breeders and researchers can integrate the Spotibot tool for fast and objective screening of rose genotypes for *Botrytis* sensitivity.

## Author contributions

DJAR: manuscript writing, software design, model training, conceptualization. MZ: manuscript writing, data collection, model training, conceptualization. MM: Data collection. PA, SG & RGFV: review manuscript, supervision, conceptualization, grant proposition.

## Data availability

Spotibot © 2024 by Wageningen University & Research is licensed under Creative Commons Attribution-ShareAlike 4.0 International. To view a copy of this license, visit https://creativecommons.org/licenses/by-sa/4.0/. Source code and algorithm of Spotibot are distributed under the aforementioned license, requiring reusers to give credit to the creator. It allows reusers to distribute, remix, adapt, and build upon the material in any medium or format, even for commercial purposes. If others remix, adapt, or build upon the material, they must license the modified material under identical terms.

The test dataset used and analyzed in the current study is available in the following link: https://figshare.com/articles/dataset/Spotibot_Rose_/28163228 while the rest of the dataset can be obtained from the corresponding author upon reasonable request. The QR codes in the dataset provided are covered to ensure that plant breeding information remains confidential.

## Funding

This work is part of a public-private-partnership project called “Variation in S gene and S gene dosage to increase *Botrytis* resistance in rose and strawberry” (Project number: LWV21.027) funded by the Dutch Topsector for Knowledge and Innovation and co-funded by the corresponding industry partners Dümmen Orange, De Ruiter Innovations, Interplant, Meilland International and United Selections.

## Declaration of competing interest

The authors declare that they have no known competing financial interests or personal relationships that could have appeared to influence the work reported in this paper.

## Abbreviations

ANOVA: Analysis of Variance
CPU: Central Processing Unit
EXIF: Exchangeable Image File
*FN*: False Negative
*FP*: False Positive
GELAN: Generalized Efficient Layer Aggregation Network
GPU: Graphical Processing Unit
HSD: (Tukey's) Honestly Significant Difference
HSV: Hue Saturation Value
*IoU*: Intersection over Union
PDA: Potato Dextrose Agar
PGI: Programmable Gradient Information
RAM: Random-access Memory
RGB: Red Blue Green
*TP*: True Positive
YOLO: You Only Look Once
