# Supplementary material for: Spotibot: Rapid scoring of Botrytis lesions on rose petals using deep learning and mobile computing
Source: Plant Phenomics. 2025 Mar 19;7(2):100029. doi: 10.1016/j.plaphe.2025.100029 (PMC12709940; doi:10.1016/j.plaphe.2025.100029)
Supplement: Multimedia component 1 [file mmc1.docx]

Supplementary Materials

Fig. S1. Subjective scoring scale for rose *Botrytis* lesions


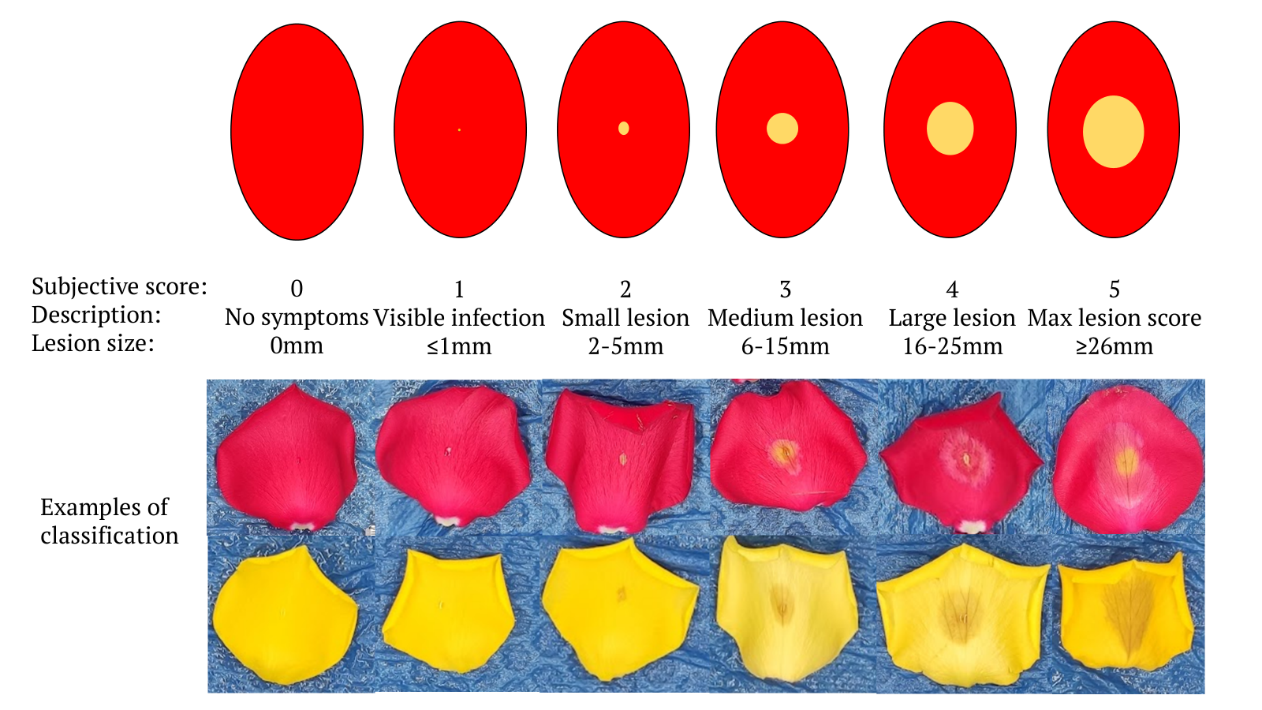


Table S1. ANOVA testing results on the entire dataset comparing 212 genotypes, where objective phenotyping means using Spotibot

| Dependent variable | Phenotyping method | Independent variable | Df | Sum Sq . | *Mean Sq .* | F value | Pr(>F) |
| --- | --- | --- | --- | --- | --- | --- | --- |
| Diameter AUDPC | Objective | Genotype | 211 | 3.17 x 10^9^ | 1.50 x 10^5^ | 51.46 | < 2.2 × 10^-16^ |
| Residual |  |  | 16709 | 4.88 x 10^7^ | 2.92 x 10^3^ |  |  |
| Area AUDPC | Objective | Genotype | 211 | 3.89 x 10^10^ | 1.84 x 10^8^ | 57.70 | < 2.2 × 10^-16^ |
| Residual |  |  | 16709 | 5.33 x 10^10^ | 3.19 x 10^6^ |  |  |
| Ratio AUDPC | Objective | Genotype | 211 | 9.85 x 10^6^ | 4.67 x 10^1^ | 57.93 | < 2.2 × 10^-16^ |
| Residual |  |  | 16709 | 1.35 x 10^4^ | 8.10 x 10^-1^ |  |  |
| Score AUDPC | Subjective | Genotype | 211 | 2.39 x 10^6^ | 1.39 x 10^3^ | 41.74 | < 2.2 × 10^-16^ |
| Residual |  |  | 16709 | 5.56 x 10^5^ | 3.33 x 10^1^ |  |  |
| Lesion diameter at 3DPI | Objective | Genotype | 211 | 1.23 x 10^6^ | 5.84 x 10^3^ | 41.28 | < 2.2 × 10^-16^ |
| Residual |  |  | 16709 | 2.36 x 10^6^ | 1.41 x 10^2^ |  |  |
| Lesion area at 3DPI | Objective | Genotype | 211 | 9.23 x 10^8^ | 4.38 x 10^6^ | 35.37 | < 2.2 × 10^-16^ |
| Residual |  |  | 16709 | 2.07 x 10^9^ | 1.24 x 10^5^ |  |  |
| Ratio at 3DPI | Objective | Genotype | 211 | 3.70 x 10^2^ | 1.75 x 10^0^ | 46.21 | < 2.2 × 10^-16^ |
| Residual |  |  | 16709 | 6.34 x 10^2^ | 4.00 x 10^-2^ |  |  |
| Score at 3DPI | Subjective | Genotype | 211 | 1.40 x 10^4^ | 6.63 x 10^1^ | 40.51 | < 2.2 × 10^-16^ |
| Residual |  |  | 16709 | 2.73 x 10^4^ | 1.64 x 10^0^ |  |  |
| Lesion diameter at 6DPI | Objective | Genotype | 211 | 2.92 x 10^6^ | 1.39 x 10^4^ | 44.70 | < 2.2 × 10^-16^ |
| Residual |  |  | 16709 | 5.18 x 10^6^ | 3.10 x 10^2^ |  |  |
| Lesion area at 6DPI | Objective | Genotype | 211 | 6.33 x 10^9^ | 3.00 x 10^7^ | 56.75 | < 2.2 × 10^-16^ |
| Residual |  |  | 16709 | 8.84 x 10^9^ | 5.29 x 10^5^ |  |  |
| Ratio at 6DPI | Objective | Genotype | 211 | 1.08 x 10^3^ | 5.09 x 10^0^ | 42.40 | < 2.2 × 10^-16^ |
| Residual |  |  | 16709 | 2.01 x 10^3^ | 1.20 x 10^-1^ |  |  |
| Score at 6DPI | Subjective | Genotype | 211 | 1.79 x 10^4^ | 8.48 x 10^1^ | 33.41 | < 2.2 × 10^-16^ |
| Residual |  |  | 16709 | 4.24 x 10^4^ | 2.54 x 10^0^ |  |  |
